# Effect of 1-methyl-1,2,3,4-tetrahydroisoquinoline on the protective action of various antiepileptic drugs in the maximal electroshock-induced seizure model: a type II isobolographic analysis

**DOI:** 10.1007/s00702-013-1047-4

**Published:** 2013-06-07

**Authors:** Marta Andres-Mach, Anna Zadrożniak, Agnieszka Haratym-Maj, Magdalena Florek-Luszczki, Grzegorz Raszewski, Lucyna Antkiewicz-Michaluk, Jarogniew J. Luszczki

**Affiliations:** 1 Isobolographic Analysis Laboratory, Institute of Rural Health, Jaczewskiego 2, 20-950 Lublin, Poland; 2 Department of Pathophysiology, Medical University of Lublin, Jaczewskiego 8, 20-090 Lublin, Poland; 3 Department of Physiopathology, Institute of Rural Health, Jaczewskiego 2, 20-950 Lublin, Poland; 4 Department of Public Health, Institute of Rural Health, Jaczewskiego 2, 20-950 Lublin, Poland; 5 Department of Neurochemistry, Institute of Pharmacology, Polish Academy of Science, Smetna 12, 31-343 Krakow, Poland

**Keywords:** 1-MeTHIQ, Antiepileptic drugs, MES-induced seizures, Pharmacokinetic/pharmacodynamic interaction

## Abstract

The aim of this study was to characterize the interaction between 1-methyl-1,2,3,4-tetrahydroisoquinoline (1-MeTHIQ—an endogenous parkinsonism-preventing substance) and various antiepileptic drugs [AEDs: clonazepam (CZP), ethosuximide (ETS), gabapentin (GBP), levetiracetam (LEV), tiagabine (TGB) and vigabatrin (VGB)] in the mouse maximal electroshock (MES)-induced seizure model. Results indicate that 1-MeTHIQ in combination with CZP (at the fixed ratios of 50:1 and 25:1), ETS (1:10) and GBP (1:1, 1:2, 1:5 and 1:10) exerted supra-additive (synergistic) interactions in the mouse MES model. In contrast, 1-MeTHIQ in combination with CZP (200:1 and 100:1), ETS (1:1, 1:2 and 1:5), LEV and VGB (1:1, 1:2, 1:5 and 1:10), and TGB (200:1, 100:1, 50:1 and 25:1) produced additive interaction in the mouse MES model. Total brain AED concentrations were unaffected by 1-MeTHIQ, and inversely, CZP, ETS and GBP had no impact on total brain concentrations of 1-MeTHIQ, indicating pharmacodynamic nature of synergistic interactions between 1-MeTHIQ and the tested AEDs in the mouse MES model. In conclusion, the supra-additive interactions of 1-MeTHIQ with CZP (at the fixed ratios of 50:1 and 25:1), ETS (1:10) and GBP (1:1, 1:2, 1:5 and 1:10) in the mouse MES model appear to be particularly favorable combinations from a clinical viewpoint. The additive combinations of 1-MeTHIQ with CZP (100:1, 50:1), ETS (1:1, 1:2 and 1:5), LEV and VGB (1:1, 1:2, 1:5, and 1:10), and TGB (200:1, 100:1, 50:1 and 25:1) seem to be neutral and worthy of consideration in further clinical practice.

## Introduction

1-Methyl-1,2,3,4-tetrahydroisoquinoline (1-MeTHIQ) is present in the human and rodent brains as a mixture of stereospecific (*R*)- and (*S*)-1-MeTHIQ enantiomers (Antkiewicz-Michaluk et al. 2011). The racemate (*R*,*S*)-1-MeTHIQ, exhibits neuroprotective activity and plays a crucial physiological role in the mammalian brain as an endogenous regulator of dopaminergic activity (Antkiewicz-Michaluk et al. 2001, 2003, 2004, 2011). 1-MeTHIQ is an endogenous amine synthesized enzymatically by 1-MeTHIQ-ase (a membrane-bound protein) localized in the mitochondrial—synaptosomal fraction (Yamakawa and Ohta 1997; Yamakawa et al. 1999). This naturally occurring substance displayed neuroprotective effects against various experimental neurotoxins, including 1-methyl-4-phenyl-1,2,3,6-tetrahydropyridine, 1-methyl-4-phenylpyridinium, beta-carbolines, tetrahydroisoquinoline, 1-benzyl-1,2,3,4-tetrahydroisoquinoline and rotenone (Antkiewicz-Michaluk et al. 2003, 2004; Kotake et al. 1995, 1996, 2005; Tasaki et al. 1991; Yamakawa and Ohta 1997, 1999; Yamakawa et al. 1999).

A large body of evidence suggests that 1-MeTHIQ offers a unique and complex mechanism of neuroprotection, in which antagonism to the glutamatergic system may play a very important role, suggesting a potential of 1-MeTHIQ as a therapeutic agent in various neurodegenerative disorders of the central nervous system (Antkiewicz-Michaluk et al. 2006). In vitro studies have revealed that 1-MeTHIQ antagonized a rise in brain dopamine metabolism and glutamate release in frontal cortex (Pietraszek et al. 2009). 1-MeTHIQ shares neuroprotective abilities with established uncompetitive NMDA receptor antagonists, which indirectly suggests that the inhibitory effect of 1-MeTHIQ on NMDA receptors plays a key role in its anti-excitotoxic activity and 1-MeTHIQ-evoked neuroprotection seems to be evoked by induction of neuronal tolerance to excitotoxicity (Kuszczyk et al. 2010).

Recently, 1-MeTHIQ has been examined as a potential anticonvulsant agent in one of the experimental models of primarily generalized seizures, the maximal electroshock (MES)-induced seizure model in mice (Luszczki et al. 2006a, 2009, 2010). The MES-induced seizures are thought to be an experimental model of tonic–clonic seizures and, to a certain extent, of partial convulsions with or without secondary generalization in man (Löscher et al. 1991; Löscher 2002). Results obtained previously indicate that 1-MeTHIQ elevated the threshold for electroconvulsions in mice (Luszczki et al. 2006a), and exerted the anticonvulsant action in mice at times ranging between 5 and 120 min after its systemic (i.p.) administration (Luszczki et al. 2009). Additionally, 1-MeTHIQ enhanced the protective action of carbamazepine (CBZ) and valproate (VPA), but not that of phenobarbital (PB) or phenytoin (PHT) against MES-induced seizures in mice (Luszczki et al. 2006a). Moreover, the isobolographic analysis of interaction revealed that 1-MeTHIQ synergistically interacted with PB and exerted the additive interaction when concomitantly administered with CBZ, PHT and VPA in the mouse MES model (Luszczki et al. 2009). With type I isobolographic analysis, the combination of 1-MeTHIQ with topiramate (TPM) exerted supra-additive (synergistic) interaction, whereas the combination of 1-MeTHIQ with oxcarbazepine (OXC), lamotrigine (LTG) and pregabalin (PGB) produced additive interaction in the mouse MES model (Luszczki et al. 2010). Generally, the second-generation antiepileptic drugs (AEDs) are known to possess a wider spectrum of anticonvulsant activity, lower risk of side effects or toxicity in comparison to classical AEDs, which definitely predispose this group of drugs to more advanced experimental and clinical studies.

In the present study, we decided to continue the experiments using 1-MeTHIQ in combination with six various AEDs: clonazepam (CZP), ethosuximide (ETS), gabapentin (GBP), levetiracetam (LEV), tiagabine (TGB) and vigabatrin (VGB) in the mouse MES model using type II isobolographic analysis. These AEDs (except for CZP and ETS) are clinically used in patients with partial seizures with or without secondary generalization (Brodie and Schachter 2001). In case of CZP, the drug is primarily used in myoclonic and atonic seizures, whereas ETS is used in patients with absence seizures (Brodie and Schachter 2001). Of note, experimental evidence indicates that the studied AEDs (i.e., CZP, ETS, LEV and TGB) are “virtually ineffective” in the mouse MES model, because the median toxic doses (TD_50_ values) of these drugs were considerably lower than their corresponding ED_50_ values as determined in the mouse MES model (White et al. 2002). Similarly, GBP has a truncated ED_50_ value in the mouse MES model and thus, it was considered as virtually ineffective in this seizure model (Dalby and Nielsen 1997). In case of VGB, we have documented in our pilot study that the drug applied i.p. at doses up to 800 mg/kg did not protect any animals against MES-induced seizures. This is the reason that we accepted that CZP, ETS, GBP, LEV, TGB and VGB are virtually inactive in the mouse MES-induced seizure model, which qualifies them to be used in the type II isobolographic analysis.

This study evaluates a potential use of 1-MeTHIQ as an AED, especially when combined with various AEDs. This could be an interesting treatment option when considering the fact that 1-MeTHIQ is an endogenous molecule present in the brain. Additionally, acute adverse effects produced by the drugs in combination were assessed using the chimney (motor performance) and grip-strength (skeletal muscular strength) tests. For the drug combinations that produced supra-additive (synergistic) interactions with 1-MeTHIQ, total brain concentrations of 1-MeTHIQ and AEDs were measured to determine any pharmacokinetic contribution to the observed anticonvulsant effects.

## Materials and methods

### Animals and experimental conditions

All experiments were performed on adult male albino Swiss mice (weighing 22–26 g, 7-week old) purchased from a licensed breeder (J. Kolacz, Warszawa, Poland). The mice were kept in colony cages with free access to food and tap water under standardized housing conditions (12 h/12 h light–dark cycle, light on 6:00 a.m., temperature of 21 ± 1 °C, relative humidity of 55 ± 3 %). After 7 days of adaptation to laboratory conditions, the animals were randomly assigned to experimental groups consisting of 8 mice. Each mouse was used only once. All tests were performed between 9.00 a.m. and 2.00 p.m. Procedures involving animals and their care were conducted in accordance with the Guide for the Care and Use of Laboratory Animals as adopted and promulgated by the National Institutes of Health. Additionally, all efforts were made to minimize animal suffering and to use only the number of animals necessary to produce reliable scientific data. The experimental protocols and procedures described hereupon were approved by the First Local Ethics Committee at the Medical University in Lublin (License no. 57/2009) and Second Local Ethics Committee at the University of Life Science of Lublin (License no. 45/2010).

### Drugs

The following drugs were used: CZP (Polfa, Warszawa, Poland), ETS (Sigma, St. Louis, MO, USA), GBP (Parke-Davis GmbH, Freiburg, Germany), LEV (UCB Pharma, Braine-l’Alleud, Belgium), TGB (Sanofi Winthrop, Gentilly, France), VGB (Marion Merrell S.A., Puteaux, France), 1-MeTHIQ (gift from Dr. J. Boksa, Institute of Pharmacology, Polish Academy of Sciences, Krakow, Poland). 1-MeTHIQ was dissolved in 0.9 % NaCl, whereas the AEDs were suspended in 1 % solution of Tween-80 (Sigma, St. Louis, MO, USA) in sterile saline and administered i.p. in a volume of 5 ml/kg body weight. The control animals received adequate volume of 0.9 % NaCl and 1 % solution of Tween-80 in sterile saline. Fresh drug solutions were administered as follows: 1-MeTHIQ at 5 min (Luszczki et al. 2006a, 2009, 2010), CZP and TGB at 15 min (Dudra-Jastrzebska et al. 2009; Luszczki et al. 2003), ETS at 45 min (Dudra-Jastrzebska et al. 2009), LEV and GBP at 60 min (Luszczki et al. 2012), and VGB at 240 min (Luszczki et al. 2005), prior to MES, chimney and grip-strength tests, as well as, before brain sampling for the measurement of 1-MeTHIQ and AED concentrations. To minimize handling effects on animals’ behavior, each mouse received two injections, including the control group of animals. The times to the peak of maximum anticonvulsant effects for all AEDs were used as the reference times in all behavioral tests.

### Maximal electroshock seizure test

The protective activity of 1-MeTHIQ administered alone and six AEDs (CZP, ETS, GBP, LEV, TGB and VGB) administered in combination with 1-MeTHIQ was evaluated and expressed as median effective doses (ED_50_ in mg/kg), protecting 50 % of mice against MES-induced tonic seizures. Electroconvulsions were produced by a current (fixed current intensity of 25 mA, maximum stimulation voltage of 500 V, 50 Hz, 0.2 s stimulus duration) delivered via standard auricular electrodes by a Hugo Sachs generator (Rodent Shocker, type 221, Freiburg, Germany). The criterion for the occurrence of seizure activity was the tonic hindlimb extension. The animals were administered with different drug doses so as to obtain a variable percentage of protection against MES-induced tonic seizures. In the present study, to determine the ED_50_ value of 1-MeTHIQ, the compound was administered at doses ranging between 30 and 70 mg/kg. The anticonvulsant activity of the mixture of 1-MeTHIQ with the studied AEDs at the fixed ratios of 200:1, 100:1, 50:1 and 25:1 (for the combinations of 1-MeTHIQ with CZP and TGB), 1:1, 1:2, 1:5 and 1:10 (for the combinations of 1-MeTHIQ with ETS, GBP, LEV and VGB) was evaluated and expressed as median effective doses (ED_50__exp_ values) against MES-induced seizures. In the present study, the AEDs were administered i.p. at the following dose ranges: 1-MeTHIQ, 25–70 mg/kg; CZP, 0.1–2 mg/kg; ETS, 30–350 mg/kg; GBP, 30–150 mg/kg; LEV, 30–450 mg/kg; TGB, 0.1–2 mg/kg and VGB, 30–450 mg/kg. This experimental procedure has been described in detail in our earlier studies (Luszczki et al. 2006a, b, 2009, 2012).

### Type II isobolographic analysis of interaction

Isobolographic analysis of interaction is a mathematical method allowing the precise characterization of interactions between drugs in both preclinical and clinical studies (Berenbaum 1989). This method allows determining the interactions between drugs applied at various doses. Of note, proportions of drugs used in two-drug mixture should be constant and determined before experimental verification of interactions (Tallarida 2000). This is the reason that several fixed ratio combinations were tested isobolographically for each AED combination. In this study, we selected four standard fixed ratio combinations of 1:1, 1:2, 1:5 and 1:10 (for the combinations of 1-MeTHIQ with ETS, GBP, LEV and VGB) and four standard fixed ratio combinations of 200:1, 100:1, 50:1 and 25:1 (for the combinations of 1-MeTHIQ with CZP and TGB), which are routinely used when testing interactions with type II isobolographic analysis. To perform the isobolographic analysis of the interactions among 1-MeTHIQ and CZP, ETS, GBP, LEV, TGB and VGB (as regards their anticonvulsant activities against tonic seizures), the AEDs in numerous fixed ratio combinations were administered to animals. Subsequently, the experimentally derived ED_50 exp_ values (±SEM) for the mixture were determined using log-probit analysis (Litchfield and Wilcoxon 1949). Moreover, theoretically additive ED_50 add_ values (±SEM) were calculated from the equation presented by Porreca et al. (1990), as follows: ED_50 add_ = ED_50 drug 1_/*P*_1_, where *P*_1_ is the proportion of the first drug, fully effective against tonic seizures (1-MeTHIQ) in the total amount of two-drug mixture. It should be noted that for two-drug mixtures, the equation presented above is true when *P*_1_ + *P*_2_ = 1, where *P*_2_ is the proportion of the second drug, virtually ineffective in the MES-induced seizure test (i.e., CZP, ETS, GBP, LEV, TGB and VGB). The proportions of AEDs in the mixture are based on a mass quantity of AEDs (for instance, a fixed ratio combination of 1:1 comprised equal amounts of 1-MeTHIQ and an AED). This particular kind of type II isobolographic analysis allows the acceptance of mass quantity of drugs in the mixture as a basis to construct the notation of fixed ratio combinations. For instance, for the fixed ratio of 1:2 for 1-MeTHIQ + ETS combination, the proportion of 1-MeTHIQ was 1/3 = 0.3333, while the proportion of ETS was 2/3 = 0.6666, in the total amount of the mixture. Subsequently, the theoretical amount of pure additive (ED_50 add_) mixture for the fixed ratio of 1:2 is calculated as follows: ED_50_ of 1-MeTHIQ divided by *P*_1_. Hence, ED_50 add_ = 48.61/0.3333 = 145.83 mg/kg (Table 1). On the other hand, the fixed ratio of 1:2 provides us with information that the second drug used in the mixture (a virtually ineffective AED) is administered at doses two times higher than that for the first fully effective drug in the mixture. A more detailed description and the theoretical background relating to isobolographic analysis including equations to calculate ED_50 add_ values and their SEM have been presented in our previous studies (Luszczki et al. 2006b, 2009). Finally, to determine the separate doses of 1-MeTHIQ and CZP, ETS, GBP, LEV, TGB and VGB in the mixture, the ED_50 exp_ values were multiplied by the respective proportions of AEDs (denoted for purely additive mixture).

**Table 1:** Isobolographic characterization of interactions between 1-MeTHIQ and the various AEDs in the mouse MES-induced seizure model

Drug combination	Fixed ratio	1-MeTHIQ_exp_	Drug_exp_	ED_50 exp_	*n*_exp_	ED_50 add_	*n*_add_	Drug_add_	1-MeTHIQ_add_
1-MeTHIQ + CZP	200:1	45.54	0.23	45.77 ± 4.94	24	48.85 ± 5.37	22	0.24	48.61
1-MeTHIQ + CZP	100:1	42.28	0.42	42.70 ± 4.76	8	49.09 ± 5.40	22	0.48	48.61
1-MeTHIQ + CZP	50:1	33.66	0.67	34.33 ± 2.71*	16	49.56 ± 5.45	22	0.95	48.61
1-MeTHIQ + CZP	25:1	31.09	1.24	32.33 ± 1.54**	24	50.48 ± 5.55	22	1.87	48.61
1-MeTHIQ + ETS	1:1	43.95	43.95	87.90 ± 3.82	16	97.22 ± 10.69	22	48.61	48.61
1-MeTHIQ + ETS	1:2	44.73	89.47	134.20 ± 16.48	16	145.83 ± 16.04	22	97.22	48.61
1-MeTHIQ + ETS	1:5	38.02	190.08	228.10 ± 27.41	24	291.66 ± 32.07	22	243.05	48.61
1-MeTHIQ + ETS	1:10	31.53	315.25	346.78 ± 23.83**	16	534.71 ± 58.80	22	486.10	48.61
1-MeTHIQ + GBP	1:1	25.83	25.83	51.66 ± 5.85***	32	97.22 ± 10.69	22	48.61	48.61
1-MeTHIQ + GBP	1:2	21.78	43.56	65.34 ± 7.12***	16	145.83 ± 16.04	22	97.22	48.61
1-MeTHIQ + GBP	1:5	14.42	72.12	86.54 ± 12.95***	24	291.66 ± 32.07	22	243.05	48.61
1-MeTHIQ + GBP	1:10	13.04	130.36	143.40 ± 19.33***	16	534.71 ± 58.80	22	486.10	48.61
1-MeTHIQ + LEV	1:1	49.33	49.33	98.66 ± 8.58	24	97.22 ± 10.69	22	48.61	48.61
1-MeTHIQ + LEV	1:2	47.89	95.79	143.68 ± 14.56	32	145.83 ± 16.04	22	97.22	48.61
1-MeTHIQ + LEV	1:5	40.20	201.02	241.22 ± 24.31	32	291.66 ± 32.07	22	243.05	48.61
1-MeTHIQ + LEV	1:10	40.01	400.12	440.13 ± 25.25	32	534.71 ± 58.80	22	486.10	48.61
1-MeTHIQ + TGB	200:1	53.84	0.27	54.11 ± 4.75	16	48.85 ± 5.37	22	0.24	48.61
1-MeTHIQ + TGB	100:1	44.84	0.45	45.29 ± 4.57	24	49.09 ± 5.40	22	0.48	48.61
1-MeTHIQ + TGB	50:1	41.08	0.82	41.90 ± 2.59	16	49.56 ± 5.45	22	0.95	48.61
1-MeTHIQ + TGB	25:1	39.37	1.58	40.94 ± 3.35	24	50.48 ± 5.55	22	1.87	48.61
1-MeTHIQ + VGB	1:1	37.68	37.68	75.36 ± 4.96	24	97.22 ± 10.69	22	48.61	48.61
1-MeTHIQ + VGB	1:2	42.80	85.59	128.39 ± 9.26	24	145.83 ± 16.04	22	97.22	48.61
1-MeTHIQ + VGB	1:5	40.18	200.91	241.09 ± 21.02	24	291.66 ± 32.07	22	243.05	48.61
1-MeTHIQ + VGB	1:10	40.18	401.81	441.99 ± 38.53	24	534.71 ± 58.80	22	486.10	48.61

Data are presented as median effective doses (ED_50_ in mg/kg ± SEM) protecting 50 % of animals tested against MES-induced seizures. ED_50_ values were either experimentally determined from the mixture of two AEDs (ED_50 exp_) or theoretically calculated from the equation of additivity (ED_50 add_). Statistical evaluation of the data was performed using the unpaired Student’s *t* test with Welch’s correction. 1-MeTHIQ_exp_, Drug_exp_, 1-MeTHIQ_add_, Drug_add_ are particular doses of 1-MeTHIQ and various AEDs in experimental (_exp_) and additive (_add_) mixture; *n* the total number of animals at those doses whose expected anticonvulsant effects ranged between 4 and 6 probits, denoted for the experimental mixture of drugs (*n*_exp_) and theoretically calculated (*n*_add_) from the equation of additivity

* *P* < 0.05, ** *P* < 0.01 and *** *P* < 0.001 versus the respective ED_50 add_, indicating supra-additive (synergistic) interaction

### Measurement of total brain concentrations of 1-MeTHIQ and AEDs

Pharmacokinetic evaluation of total brain concentrations of 1-MeTHIQ and AEDs was performed only for those combinations of 1-MeTHIQ with AEDs that produced synergistic interaction in the mouse MES test. Thus, the measurement of total brain concentrations of 1-MeTHIQ and CZP, ETS and GBP was undertaken at the drug doses corresponding to the fixed ratios of 25:1 (1-MeTHIQ + CZP) and 1:10 (1-MeTHIQ + ETS and 1-MeTHIQ + GBP) from the MES test, respectively. Mice were killed by decapitation at times chosen to coincide with that scheduled for the MES test and whole brains were removed from skulls, weighed, harvested and homogenized using Abbott buffer (2:1, v/w; Abbott Laboratories, North Chicago, IL, USA) in an Ultra-Turrax T8 homogenizer (IKA Werke, Staufen, Germany). The brain homogenates were centrifuged at 10,000×*g* for 10 min and the supernatant samples were analyzed either by fluorescence polarization immunoassay (FPIA: CZP and ETS) or by high-performance liquid chromatography (HPLC: 1-MeTHIQ and GBP). The supernatant samples (75 μl) were analyzed by FPIA for CZP and ETS content using a TDx analyzer and reagents as described by the manufacturer (Abbott Laboratories, North Chicago, IL, USA). Control samples of CZP and ETS were placed at the beginning and end of each carousel for verification of the calibration. For the quantitation of CZP, the benzodiazepine assay kit was used. Total brain concentrations of 1-MeTHIQ were determined by HPLC using a Dionex HPLC system (Sunnyvale, CA, USA) comprising a quaternary pump P 580, vacuum degasser and UV–vis detector (UVD 340S). The mobile phase consisting of 0.08 M triethylammonium phosphate buffer solution (pH 3.6) and methanol in the ratio of 85:15 (v/v) and chromatographic separation was achieved using a Zorbax SB-C18 (5 μm) column (Agilent Technologies, Santa Clara, CA, USA). Chromatography was performed at ambient temperature using a flow ratio of 1.2 ml/min. The column eluate was monitored at 215 nm with a sensitivity of 0.01 absorbance unit full scale (AUFS). To detect 1-MeTHIQ concentrations, brain homogenate samples were prepared for analysis as follows: 200 μl brain homogenate was pipetted into 2.5 ml plastic centrifugal filter devices (Millipore Corporation, Billerica, MA, USA), to which 200 μl of 0.08 M triethylammonium phosphate buffer solution and 400 μl methanol were added and vortex-mixed for 1 min. After centrifugation (at 10,000×*g* for 10 min) the organic layer was removed and 20 μl of the aqueous phase was injected into the HPLC system. Quantitation was achieved using chromatographic peak height and that was linearly related over the range of 0.4–20 μg/ml of 1-MeTHIQ. The within-batch and between-batch precision was <8 and <7 %, respectively. Total brain concentrations of GBP were determined by HPLC using a Dionex HPLC system (Sunnyvale, CA, USA) comprising a quaternary pump P 580, vacuum degasser and UV–vis detector (UVD 340S). The mobile phase consisted of 50 mM sodium phosphate monobasic buffer (pH 2.5), methanol and acetonitrile in the ratio of 45:40:12 (v/v) and chromatographic separation was achieved using Hibar 125-4 Li-Chrosorb RP-8 (5 μm) column (Merck Millipore, Darmstadt, Germany). Chromatography was performed at ambient temperature using a flow ratio of 1.0 ml/min. The column eluate was monitored at 254 nm with a sensitivity of 0.01 AUFS. To detect GBP concentrations, brain homogenate samples were prepared for analysis as follows: 200 μl brain homogenate was pipetted into 2.5 ml plastic centrifugal filter devices (Millipore Corporation, Billerica, MA, USA), to which 50 μl of internal standard (baclofen, 30 μl/ml) and 400 μl of acetonitrile were added and vortex-mixed for 5 min. After centrifugation at 12,000×*g* for 10 min, 2 ml of mixture of dichloromethane:2-propanol (1:1) was added and subsequently centrifuged at 4,000×*g* for 15 min. 0.1 M NaOH was added to the eluent and the mixture was evaporated to dryness. 150 μl of derivatizing reagent (0.35 % phenylisothiocyanate in methanol) was added to dissolve and react with the residue. The reaction took place at room temperature. The reaction mixture was then evaporated to dryness. The residue was reconstituted with 250 μl of acetonitrile and 20 μl was injected into the HPLC system for analysis. Quantitation was achieved using chromatographic peak height and that was linearly related over the range of 1–100 μg/ml of GBP. The within-batch and between-batch precision was <5 and <6 %, respectively. Total brain concentrations of 1-MeTHIQ, ETS and GBP were expressed in μg/ml of brain supernatants, whereas those of CZP were expressed in ng/ml of brain supernatants as mean ± SEM of at least 8 separate brain preparations.

### Chimney test

The effects of 1-MeTHIQ and the studied AEDs (CZP, ETS, GBP, LEV, TGB and VGB) administered in combination with 1-MeTHIQ (at doses corresponding to their ED_50 exp_ values from the MES-induced seizure test) on motor coordination impairment were quantified with the chimney test of Boissier et al. (1960). In the chimney test, animals had to climb backwards up the plastic tube (3 cm inner diameter, 30 cm length). Motor impairment was indicated by the inability of the animals to climb backward up the transparent tube within 60 s. Data were presented as a percentage of animals that failed to perform the chimney test. This experimental procedure has been described in detail in our earlier studies (Luszczki et al. 2005, 2006a).

### Grip-strength test

The effects of 1-MeTHIQ and the studied AEDs in combination with 1-MeTHIQ (administered at doses corresponding to their ED_50 exp_ values from the MES-induced seizure test) on skeletal muscular strength in mice were quantified by the grip-strength test of Meyer et al. (1979). The grip-strength apparatus (BioSeb, Chaville, France) comprised a wire grid (8 cm × 8 cm) connected to an isometric force transducer (dynamometer). The mice were lifted by the tails so that their forepaws could grasp the grid. The mice were then gently pulled backward by the tail until the grid was released. The maximal force exerted by the mouse before losing grip was recorded. The mean of three measurements for each animal was calculated and subsequently, the mean maximal force of eight animals per group was determined. The skeletal muscular strength in mice was expressed in N (newton) as mean ± SEM of at least eight determinations. This experimental procedure has been described in detail in our earlier study (Luszczki et al. 2008, 2009; Zadrozniak et al. 2009).

### Statistics

The ED_50_ value for 1-MeTHIQ and ED_50__exp_ values (with their respective 95 % confidence limits) for the combinations of 1-MeTHIQ with CZP, ETS, GBP, LEV, TGB and VGB at various fixed ratios in the MES-induced seizure test were calculated by computer-assisted log-probit analysis according to Litchfield and Wilcoxon (1949). The obtained 95 % confidence limits were transformed to SEM as described previously (Luszczki et al. 2006b, 2009). Statistical evaluation of isobolographic interactions was performed by the use of Student’s *t* test to detect the differences between the experimentally derived (ED_50 exp_) and theoretical additive (ED_50 add_) values, according to Porreca et al. (1990). The results from the grip-strength test were statistically analyzed with one-way ANOVA followed by the post hoc Bonferroni’s test for multiple comparisons. The Fisher’s exact probability test was used to analyze the results from the chimney test. Results were considered statistically significant if *P* < 0.05.

### Software used

Microsoft’s Excel spreadsheet was used to perform calculations and to graphically illustrate the results in form of isobolograms. This spreadsheet was programmed to compute all calculations automatically from the log-probit linear regression analysis according to Litchfield and Wilcoxon (1949). The theoretically additive ED_50 add_ values and their SEM at the various fixed ratio combinations were also calculated with this programmed spreadsheet. All statistical tests were performed using commercially available GraphPad Prism version 4.0 for Windows (GraphPad Software, San Diego, CA, USA).

## Results

### Effect of 1-MeTHIQ and its combination on the anticonvulsant activity of AEDs against MES-induced seizures in mice

**Fig. 1:**
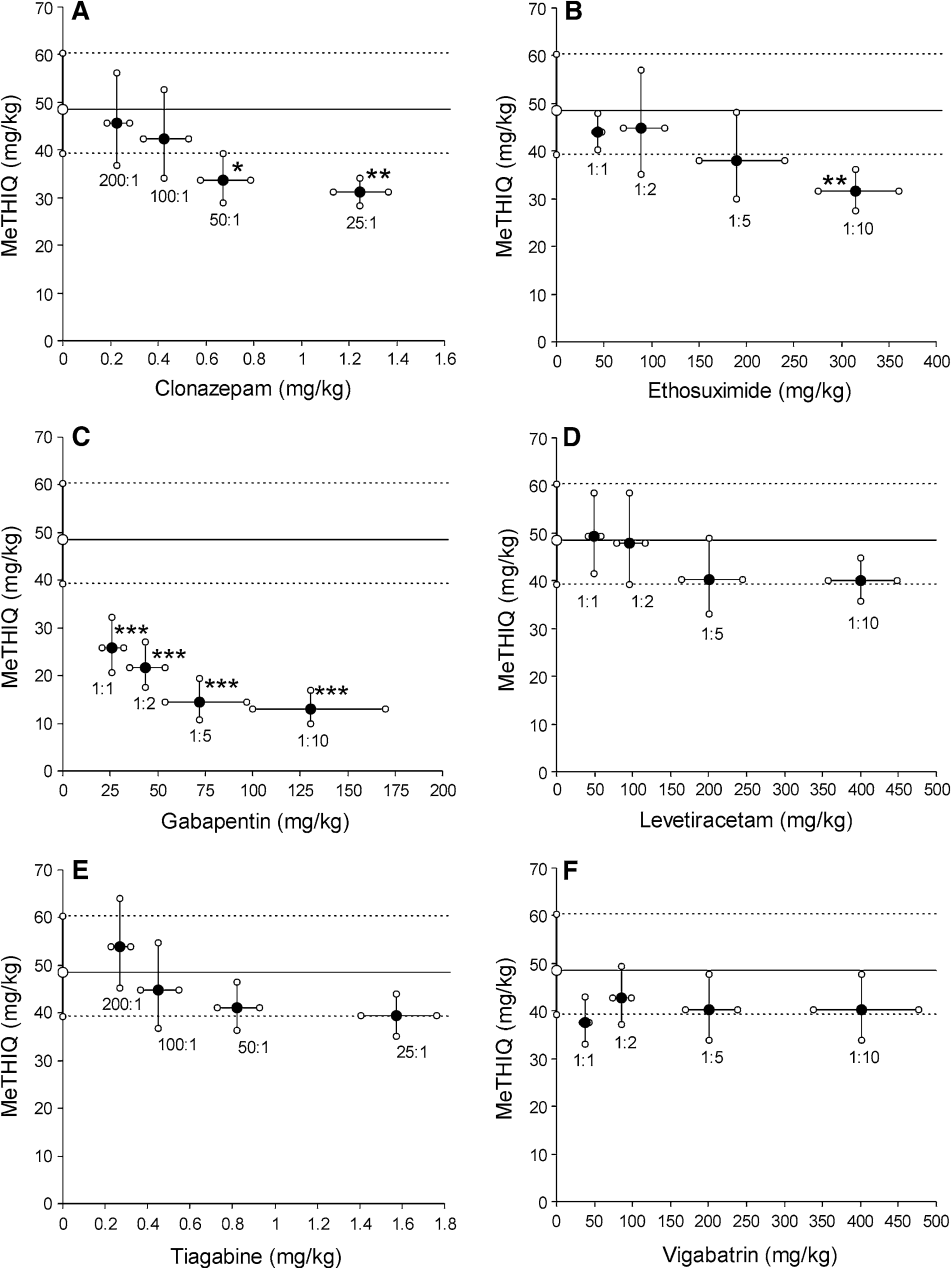
**a**–**f** Isobolograms showing interactions among 1-MeTHIQ and clonazepam (CZP), ethosuximide (ETS), gabapentin (GBP), levetiracetam (LEV), tiagabine (TGB) and vigabatrin (VGB) in the mouse MES-induced seizure model. 1-MeTHIQ doses are plotted graphically on the *Y* axis, whereas the doses of CZP, ETS, GBP, LEV, TGB and VGB are plotted on the *X* axis (**a**–**f**). The *heavy line* is parallel to the *X* axis, representing the ED_50_ value for 1-MeTHIQ administered alone, and defines the theoretical dose-additive line for a continuum of different fixed dose ratios. The *dotted lines* represent SEM values for 1-MeTHIQ administered alone. The *closed circles* depict the experimentally derived ED_50 exp_ values for total doses of mixtures expressed as proportions of 1-MeTHIQ and an AED that produced median anticonvulsant effects. The SEM represents an actual calculation of the vertical and horizontal components of the error. **a** Interactions between 1-MeTHIQ and CZP. The experimental ED_50 exp_ values for the mixture of 1-MeTHIQ + CZP for the fixed ratios of 50:1 and 25:1 are significantly below the theoretical line of additivity, indicating supra-additivity (*P < 0.05 and **P < 0.01). The fixed ratio combinations of 200:1 and 100:1 indicate additive interactions. **b** Interactions between 1-MeTHIQ and ETS. The experimental ED_50 exp_ value for the mixture of 1-MeTHIQ + ETS for the fixed ratios of 1:10 is significantly below the theoretical line of additivity, indicating supra-additivity (**P < 0.01). The fixed ratio combinations of 1:1, 1:2 and 1:5 indicate additive interactions. **c** Interactions between 1-MeTHIQ and GBP. The experimental ED_50 exp_ values of the mixture of 1-MeTHIQ + GBP for the fixed ratios of 1:1, 1:2, 1:5 and 1:10 are significantly below the theoretical line of additivity, indicating supra-additivity (***P < 0.001). **d** Interactions between 1-MeTHIQ and LEV. The experimentally derived ED_50 exp_ values of the mixture of 1-MeTHIQ + LEV for the fixed ratios of 1:1, 1:2, 1:5 and 1:10 did not significantly differ from the theoretically calculated ED_50 add_ values and thus, the observed interactions between 1-MeTHIQ and LEV are additive. **e** Interactions between 1-MeTHIQ and TGB. The experimentally derived ED_50 exp_ values of the mixture of 1-MeTHIQ + TGB for the fixed ratios of 200:1, 100:1, 50:1 and 25:1 did not significantly differ from the theoretically calculated ED_50 add_ values and thus, the observed interactions between 1-MeTHIQ and TGB are additive. **f** Interactions between 1-MeTHIQ and VGB. The experimentally derived ED_50 exp_ values of the mixture of 1-MeTHIQ + VGB for the fixed ratios of 1:1, 1:2, 1:5 and 1:10 did not significantly differ from the theoretically calculated ED_50 add_ values and thus, the observed interactions between 1-MeTHIQ and VGB are additive

1-MeTHIQ administered systemically (i.p.) exerted a clear-cut anticonvulsant action in the mouse MES model and its experimentally derived ED_50_ value was 48.61 (39.18–60.31) mg/kg. 1-MeTHIQ in combination with CZP, at the fixed ratios of 50:1 and 25:1, produced supra-additive (synergistic) interaction (Fig. 1), whereas the two-drug combinations at the fixed ratios of 200:1 and 100:1 were additive in the mouse MES model (Table 1; Fig. 1). 1-MeTHIQ in combination with ETS, at the fixed ratio of 1:10, exerted supra-additive (synergistic) interaction (Fig. 1), whereas the two-drug combinations at the fixed ratios of 1:1, 1:2 and 1:5 were additive in the mouse MES model (Table 1; Fig. 1). Similarly, the combinations of 1-MeTHIQ with TGB at the fixed ratios of 200:1, 100:1, 50:1 and 25:1 were additive in the mouse MES model (Table 1). Likewise, the combinations of 1-MeTHIQ with LEV and VGB at the fixed ratios of 1:1, 1:2, 1:5 and 1:10 were additive in the mouse MES model (Table 1; Fig. 1). Interestingly, the combinations of 1-MeTHIQ and GBP at the fixed ratios of 1:1, 1:2, 1:5 and 1:10 were supra-additive (synergistic) in the mouse MES model (Table 1; Fig. 1).

### Total brain concentrations of 1-MeTHIQ and AEDs in mice

The total brain concentrations were measured only for the combinations of 1-MeTHIQ with AEDs that displayed supra-additive interactions in the MES test. Pharmacokinetic study revealed that total brain CZP, ETS and GBP concentrations were unaffected by 1-MeTHIQ (Table 2), and inversely, CZP, ETS and GBP had no impact on total brain concentrations of 1-MeTHIQ (Table 3), indicating pharmacodynamic nature of interactions between 1-MeTHIQ and the studied AEDs.

**Table 2:** Effect of 1-MeTHIQ on total brain concentrations of AEDs

Treatment (mg/kg)	FR	Total brain concentration	
		(μg/ml)	(ng/ml)
CZP (1.24) + vehicle	–		22.18 ± 1.69
CZP (1.24) + 1-MeTHIQ (31.09)	25:1		23.07 ± 1.83
ETS (315.3) + vehicle	–	30.31 ± 6.59	
ETS (315.3) + 1-MeTHIQ (31.53)	1:10	36.09 ± 6.97	
GBP (130.36) + vehicle	–	6.71 ± 0.55	
GBP (130.36) + 1-MeTHIQ (13.04)	1:10	5.92 ± 0.51	
GBP (25.83) + vehicle	–	2.73 ± 0.80	
GBP (25.83) + 1-MeTHIQ (25.83)	1:1	3.22 ± 0.96	

Data are presented as mean ± SEM of at least 8 separate determinations. The AED concentrations were measured using either FPIA (CZP and ETS) or HPLC (GBP). Statistical evaluation of the data was performed using the unpaired Student’s *t* test. Brain tissue samples were taken at times scheduled from the MES test

*FR* fixed ratio combination, vehicle, 0.9 % NaCl, *CZP* clonazepam, *ETS* ethosuximide, *GBP* gabapentin

**Table 3:** Effect of AEDs on total brain concentrations of 1-MeTHIQ

Treatment (mg/kg)	FR	Total brain concentration (μg/ml)
1-MeTHIQ (31.09) + vehicle^a^	–	6.08 ± 0.49
1-MeTHIQ (31.09) + CZP (1.24)	25:1	6.27 ± 0.43
1-MeTHIQ (31.53) + vehicle^a^	–	6.31 ± 0.45
1-MeTHIQ (31.53) + ETS (315.3)	1:10	6.59 ± 0.49
1-MeTHIQ (13.04) + vehicle^a^	–	2.70 ± 0.42
1-MeTHIQ (13.04) + GBP (130.36)	1:10	3.02 ± 0.45
1-MeTHIQ (25.83) + vehicle^a^	–	3.70 ± 0.56
1-MeTHIQ (25.83) + GBP (25.83)	1:1	3.88 **±** 0.53

Data are presented as mean ± SEM of at least 8 separate determinations. The concentrations of 1-MeTHIQ were measured using the HPLC. Statistical evaluation of the data was performed using the unpaired Student’s *t* test. Brain tissue samples were taken at times scheduled from the MES test

*FR* fixed ratio combination, vehicle^a^, 1 % solution of Tween-80 in sterile saline, *CZP* clonazepam, *ETS* ethosuximide, *GBP* gabapentin

### Effects of 1-MeTHIQ and its combination with AEDs on skeletal muscular strength and motor performance in the grip-strength and chimney tests in mice

1-MeTHIQ administered systemically (i.p.) did not affect muscular strength or motor coordination in mice (Table 4). Moreover, for all the studied combinations of 1-MeTHIQ with CZP, ETS, GBP, LEV, TGB and VGB, neither motor coordination nor skeletal muscular strength was affected (Table 4).

**Table 4:** Effects of 1-MeTHIQ and its combination with AEDs on skeletal muscular strength and motor performance in the grip-strength and chimney tests in mice

Treatment (mg/kg)	FR	Grip-strength	Motor impairment
		(*N*)	(%)
Vehicle + vehicle^a^	–	0.888 ± 0.043	0
1-MeTHIQ (48.61) + vehicle^a^	–	0.894 ± 0.042	0
1-MeTHIQ (31.09) + CZP (1.24)	25:1	0.910 ± 0.044	25
1-MeTHIQ (31.53) + ETS (315.3)	1:10	0.865 ± 0.045	12.5
1-MeTHIQ (13.04) + GBP (130.36)	1:10	0.943 ± 0.039	0
1-MeTHIQ (40.01) + LEV (400.12)	1:10	0.911 ± 0.041	0
1-MeTHIQ (39.37) + TGB (1.58)	25:1	0.869 ± 0.044	12.5
1-MeTHIQ (40.18) + VGB (401.81)	1:10	0.865 ± 0.045	25

Results are presented as: (1) mean grip-strengths [in newton (N) ± SEM] from the grip-strength test, assessing skeletal muscular strength in mice; (2) percentage of animals showing motor coordination impairment in the chimney test in mice. Each experimental group consisted of 8 animals. Statistical analysis of data from the grip-strength test was performed with one-way ANOVA followed by the post hoc Bonferroni’s test for multiple comparisons. The Fisher’s exact probability test was used to analyze the results from the chimney test. All drugs were administered i.p. at times scheduled from the MES test and at doses corresponding to the ED_50_ and ED_50 exp_ values against MES-induced tonic seizures

*FR* fixed ratio combination, Vehicle, 0.9 % NaCl; vehicle^a^, 1 % solution of Tween-80 in sterile saline, *CZP* clonazepam, *ETS* ethosuximide, *GBP* gabapentin, *LEV* levetiracetam, *TGB* tiagabine, *VGB* vigabatrin

## Discussion

The aim of this study was to characterize the type of interactions between 1-MeTHIQ (an endogenous parkinsonism-preventing substance) and six various AEDs (CZP, ETS, GBP, LEV, TGB and VGB) in the mouse MES model using the type II isobolographic analysis. The results indicate supra-additive (synergistic) interactions between 1-MeTHIQ and CZP, ETS and GBP at some selected fixed ratio combinations (i.e., 50:1, 25:1 for 1-MeTHIQ + CZP, 1:10 for 1-MeTHIQ + ETS, and 1:10, 1:5, 1:2 and 1:1 for 1-MeTHIQ + GBP) in the mouse MES model, and they appear to be particularly favorable combinations from the clinical viewpoint. Moreover, results obtained from the estimation of total brain concentrations of 1-MeTHIQ, CZP, ETS and GBP showed no significant changes in the total brain concentrations of the tested drugs, suggesting pharmacodynamic nature of interaction between 1-MeTHIQ and the AEDs in the mouse MES model. It is noteworthy to mention that we examined only those AED combinations that offered synergistic interactions between 1-MeTHIQ and AEDs. This is the reason that the combinations of 1-MeTHIQ with CZP, ETS, and GBP were pharmacokinetically verified in our study. Of note, the concentrations of two drugs in mixture (i.e., 1-MeTHIQ and an AED) were measured to exactly characterize the type of interactions among drugs. For instance, we have reported earlier that loreclezole (LCZ, an AED) had no impact on total brain concentration of VPA, while the latter AED significantly increased total brain concentrations of LCZ and thus, the drugs in combination produced supra-additive (synergistic) interaction in the mouse MES model (Luszczki et al. 2006b). Hence, the evaluation of VPA concentrations without LCZ could provide us with incomplete data and lead to wrong conclusions. This is the reason that in our study, the estimation of both, 1-MeTHIQ and AEDs concentrations was performed. Moreover, in isobolographic studies, the estimation of AED concentrations in brains of experimental animals has been recommended as a standard procedure because concentrations of drugs affecting central nervous system should be evaluated at the site of drug action or biophase (brain tissue or cerebrospinal fluid), where the drugs exert their anticonvulsant activity. For instance, we have documented that LCZ significantly elevated the free plasma concentrations of VPA, whereas the latter drug had no impact on total plasma concentrations of LCZ in mice (Luszczki et al. 2006b). This is the reason that the estimation of AED concentrations in plasma of experimental animals may sometimes produce false information on the nature of interaction between tested drugs (for more details see Cadart et al. 2002; Luszczki et al. 2003, 2006b).

In case of combinations of 1-MeTHIQ with GBP, we tested only the supra-additive (synergistic) combinations at the fixed ratios of 1:1 and 1:10. Since 1-MeTHIQ had no impact on total brain concentrations of GBP and, inversely, GBP did not affect total brain concentrations of 1-MeTHIQ at the fixed ratios of 1:1 and 1:10, one can indirectly accept that the drugs in synergistic combinations at the fixed ratios of 1:2 and 1:5 also exerted pharmacodynamic interactions in the mouse MES model.

It should be stressed that we did not measure total brain concentrations of 1-MeTHIQ in combination with LEV, TGB and VGB because the observed interactions in the mouse MES model were barely additive. From a preclinical viewpoint, additive interactions between drugs are not as favorable as synergistic interactions that could be advantageous in patients with epilepsy. On the other hand, LEV and VGB are considered as AEDs with ideal pharmacokinetic properties, because the drugs do not bind to or do not replace other drugs from plasma proteins, do not undergo metabolic transformation in the liver, and do not inhibit or activate cytochrome P450 isoenzymes (Patsalos 2004; Patsalos and Perucca 2003). Thus, it is unlikely that 1-MeTHIQ could affect pharmacokinetic profiles of LEV and VGB, and inversely, it is impossible that the latter drugs could influence total brain concentrations of 1-MeTHIQ. In case of TGB, approx. 96 % of the drug binds to plasma proteins (Johannessen et al. 2003). In the liver, TGB undergoes oxidative metabolism by microsomal cytochrome CYP3A4 isoenzyme and subsequent conjugation with glucuronic acid (Johannessen et al. 2003). Thus, it is impossible, but not excluded, that 1-MeTHIQ and TGB could mutually affect their pharmacokinetic profiles and significantly change their total brain concentrations in experimental animals.

Results presented herein also indicated that the remaining tested combinations of 1-MeTHIQ with ETS (at the fixed ratios of 1:1, 1:2 and 1:5) and CZP (at the fixed ratios of 200:1 and 100:1) were additive in the mouse MES-induced seizure model. Additionally, all the studied combinations of 1-MeTHIQ with LEV, TGB and VGB were additive in the mouse MES-induced seizure model and seem to be neutral from a preclinical point of view.

To find out an explanation for the supra-additive interactions of 1-MeTHIQ with CZP, ETS and GBP, one should consider molecular mechanisms of action of the tested AEDs. As regards GBP, the drug has its chemical structure very similar to the inhibitory neurotransmitter gamma-aminobutyric acid (GABA), but GBP does not act through the brain GABA receptors (Taylor et al. 1998). GBP interacts with α_2_δ subunit of voltage-gated calcium channels and thus the drug reduces synaptic release of neurotransmitters and decreases postsynaptic calcium influx resulting in diminished excitation (Taylor et al. 1998). Moreover, GBP enlarges the hyperpolarization-activated cation current (Ih) (Surges et al. 2003) and agonistically affects ATP-dependent potassium (K_ATP_) channels (Bertrand et al. 2003; Cheng et al. 2006). GBP (at high concentrations) increases the activity of the GABA-synthesizing enzyme [glutamic acid decarboxylase (GAD)] and inhibits branched-chain amino acid aminotransferase (BCAA-T), which metabolizes cytosolic l-leucine, l-isoleucine, l-valine to glutamate (Goldlust et al. 1995; Hutson et al. 1998). In case of ETS, the drug binds to the inactivated state of low-threshold T-type calcium channels and selectively inhibits pathological firing without any effect on normal neuronal activity (Coulter et al. 1989; Gomora et al. 2001). Moreover, ETS decreases the calcium-activated potassium current in thalamo-cortical neurons (Coulter et al. 1989) and partially reduces the non-inactivating sodium current (Leresche et al. 1998; Crunelli and Leresche 2002). With respect to CZP mechanisms of action, it is established that benzodiazepines (including CZP) interact specifically with a benzodiazepine receptor molecule to modulate allosterically the efficiency of the inhibitory neurotransmitter GABA at GABA_A_ receptors (Haefely 1989; Macdonald 2002). Moreover, benzodiazepines (especially, diazepam) at high concentrations also block sodium channels voltage dependently, reducing high-frequency repetitive firing in cultured mammalian neurons (McLean and Macdonald 1988). The effect of diazepam to limit repetitive firing occurred at diazepam concentrations achieved in the treatment of status epilepticus and was not blocked by the benzodiazepine receptor antagonist flumazenil (Macdonald 2002). The similar effect on repetitive firing can be evoked by CZP used in high doses. CZP is classified as a partial benzodiazepine receptor agonist (Mehta and Ticku 1989; Miller et al. 1990), which in the presence of a full agonist reduces its effect acting as mixed agonist–antagonist (Haefely 1989). In case of 1-MeTHIQ, the compound inhibits the excitatory neurotransmitter glutamatergic system through the blockade of NMDA receptors as a non-competitive NMDA receptor antagonist (Antkiewicz-Michaluk et al. 2006). At present, little is known about the binding of 1-MeTHIQ to NMDA receptor subunits and more advanced neurochemical studies are required to elucidate this phenomenon.

The above-discussed mechanisms clearly indicate that synergistic interaction between 1-MeTHIQ and GBP can be explained through the synergistic cooperation of both drugs in suppression of MES-induced tonic seizures. It seems that the blockade of NMDA receptors associated with simultaneous blockade of α_2_δ subunit of high-voltage activated calcium channels produced synergistic interaction between drugs, independently on doses of both drugs used in mixture. Of note, synergistic interaction between 1-MeTHIQ and GBP was reported for the drug mixture at all the fixed ratios tested (i.e., 1:1, 1:2, 1:5 and 1:10). In case of the combination of 1-MeTHIQ with CZP, only the two-drug combination at the fixed ratios of 25:1 and 50:1 produced synergistic interaction in the mouse MES model. It seems that CZP applied in high doses (i.e., 0.67 mg/kg at the fixed ratio of 50:1 and 1.24 mg/kg at the fixed ratio of 25:1) cooperated with 1-MeTHIQ in terms of suppression of MES-induced tonic seizures in mice. Hence, the blockade of NMDA receptors by 1-MeTHIQ and enhancement of GABA-ergic inhibitory neurotransmitter system through the modulation of GABA_A_ receptors and, particularly, the blockade of sodium channels via benzodiazepine receptors considerably contribute to the suppression of MES-induced seizures in mice. In case of the combinations of 1-MeTHIQ with ETS, only the two-drug mixture at the fixed ratio of 1:10 produced synergistic interaction in the mouse MES model. In such a situation, one can conclude that ETS (administered i.p. at a high dose of 315 mg/kg that corresponded to the fixed ratio of 1:10 in the mouse MES model) cooperated with 1-MeTHIQ. Thus, it seems that the inhibition of T-type calcium channels and, particularly, sodium channels by high doses of ETS in neurons and 1-MeTHIQ-mediated blockade of NMDA receptors suppressed MES-induced tonic seizures in rodents. In contrast, 1-MeTHIQ did not enhance the anticonvulsant action of TGB, VGB and LEV in the mouse MES model. Thus, one can ascertain that the blockade of NMDA receptors mediated by 1-MeTHIQ was insufficient so as to cooperate with irreversible inhibition of GABA-transaminase activity exerted by VGB (Czuczwar and Patsalos 2001). Similarly, TGB through the inhibition of reuptake of GABA from the synaptic clefts (Czuczwar and Patsalos 2001) or LEV through the inhibition of synaptic vesicle protein (SV2A) and reduction of the excitatory neurotransmitter release (Czapinski et al. 2005) did not cooperate with the blockade of NMDA receptors evoked by 1-MeTHIQ in terms of suppression of MES-induced tonic seizures in mice.

Evaluation of potential acute adverse effects produced by the AEDs in combination with 1-MeTHIQ revealed that no significant changes in skeletal muscular strength or motor coordination were documented in experimental animals. It should be stressed that in our previous studies, we have reported that VPA in combination with TGB significantly impaired motor coordination in mice subjected to the chimney test (Luszczki et al. 2003). Additionally, we have documented that several various AEDs (including, CBZ, VPA, CZP, PB, LTG, PHT, OXC and TPM) in a dose-dependent manner reduced skeletal muscular strength in experimental animals (Luszczki et al. 2008; Zadrozniak et al. 2009). In contrast, sildenafil enhanced skeletal muscular strength in mice challenged with the grip-strength test (Nieoczym et al. 2010). It seems that the grip-strength test allows the detection of some subtle changes in muscular strength of experimental animals after administration of tested drugs that cannot be detected in the chimney or rota-rod tests. All the above-discussed facts clearly testify that the behavioral tests used in this study were sensitive enough to detect any significant changes in normal functioning of animals after administration of 1-MeTHIQ and AEDs. Since no significant changes were reported in the behavioral tests in this study, we can conclude that all the tested combinations of 1-MeTHIQ with AEDs (applied in doses from the MES test) are safe and tolerable that could be an additional argument when recommending 1-MeTHIQ in combination with AEDs as the treatment options in further clinical settings.

Finally, there has been considerable debate as to whether rational polytherapy should consist of AEDs with similar mechanisms of action, thus allowing lower doses to be prescribed so as to achieve anticonvulsant synergy but without dose-related side effects, or AEDs with different mechanisms of action so as to increase the spectrum of anticonvulsant activity and therefore enhance seizure control (Perucca 1995; Deckers et al. 2000; Schmidt 1996). In the present study, the most advantageous combinations, using the MES model, were that of 1-MeTHIQ and CZP, ETS and GBP. The fact that 1-MeTHIQ and these AEDs have various mechanisms of action may be important in relation to the observed synergy. The results from the present study confirm a hypothesis that 1-MeTHIQ had significant effect on the anticonvulsant efficacy of various AEDs, even if they were virtually ineffective in the mouse MES-induced tonic seizure model. The combinations of 1-MeTHIQ with CZP, ETS and GBP need more advanced studies, especially, in other models of epilepsy to recommend their application in further clinical settings.

## Abbreviations

AEDs: Antiepileptic drugs
1-MeTHIQ: 1-Methyl-1,2,3,4-tetrahydroisoquinoline
CZP: Clonazepam
ETS: Ethosuximide
GBP: Gabapentin
LEV: Levetiracetam
MES: Maximal electroshock-induced seizures
TGB: Tiagabine
VGB: Vigabatrin
